# Use of Anæsthetics in Rigidity of Perenæum in First Labor

**Published:** 1853-06

**Authors:** J. H. Beech

**Affiliations:** Coldwater, Mich.


ART. II. — *Use of Anæsthetics in Rigidity of Perinæum in First Labor.* By J. H. BEECH, M. D., of Coldwater, Mich.

Holding the opinion that the small experience of the general practitioner may be rendered doubly useful to himself, and of some avail, perhaps, to others, if placed in some reservoir whence many draw mental refreshment; I beg leave to submit the following to your consideration, and that of the profession, if you deem it worthy a place in the Buffalo Med. Journal:

Having a desire to exercise great caution in the employment of new remedies, I began the use of anæsthetics in obstetric practice, only in cases of unusual severity, where pain seemed rather a dangerous than a warning symptom.

At first I was extremely reluctant in admitting them in first labors, desiring to give Dame Nature a fair chance with new apparatus. A case like the following, not the first which had come under my observation, and I believe not unheard of, in the practice of most physicians, suggested the use of anæsthetics for a definite object, under certain circumstances. A young woman in first labor, had proceeded in about the usual manner, (except that there was excessive firmness of the os externum,) until the head distended the perinæum to its utmost capacity. Venesection and tartarized antimony, etc., had lent their aid; and the perinæum had been many minutes carefully supported by the hand covered by a napkin, the reluctant *os* was slowly yielding, and time seemed the only additional remedy necessary. The pain, however, was most intolerable, and my patient, although perfectly confiding, and obedient as possible to every word of caution, hitherto, suddenly threw all her energy into one effort, which tossed her from her position, and almost from the hands of her attendants. The result was, a rupture, extending from the *fourchette*

backward about two inches; the posterior commissure being about eight lines from the *raphe*. Reflection brought resolution in regard to future trials of this kind. The course of human events brought cases unlike, and at last a similar one, in nearly all respects. The extreme pain appeared to be the only bar to my prospect of success.

Ether was accordingly administered by inhalation, and to my infinite gratification, the rigid parts yielded like warmed India rubber, and the head passed almost as soon as the subtle fluid had banished the cognizance of pain. The etherization had nearly subsided before the hips escaped.

Again, a similar patient came to my charge, but the whole process of dilatation, both internal and external, had been tardy and unusually painful, although nausea and vomiting were almost constant. The usual methods had been resorted to with that success which patience bestows, until the head bore hard upon the center of the perinæum.

Several strong uterine and abdominal contractions had exhibited a force sufficient to have made an independent passage, but for the support of the hand. With each effort the perinæum seemed to yield a little, and then contract with all its power, and carry all nearly back to the starting point of last pain. The patient had nearly exhausted self-control, and although there was evidently an enlargement of the *vulva* steadily progressing, delay was dangerous, as there was a strong disposition of the parts to become dry and tender, requiring frequent artificial lubrication.

I knew the patient to be very susceptible to the influence of chloroform, and it was accordingly administered. Its effect was as soon discovered at the seat of difficulty, as by the attendant who gave it, and three or four natural contractions, in quick succession, completed the labor, with slight perception of pain.

I have exhibited both chloroform and ether, to females not primiparous, and have often thought that the external organs relaxed more readily; whereas, formerly, I had feared injury from the withdrawal of consciousness in the last stage of labor in cases like the above. But agony as effectually destroys self-command as "Morpheus" himself, and I argue thus: the sentient nerves being quieted, dame nature has the more perfect control.

If the experience of others agrees with my own thus far, anæsthetics are useful adjuvants in labor with rigidity of the soft parts, especially where it possesses the character of clonic spasm.

*April* 18, 1853.

